# Micro- and nano-surface structures based on vapor-deposited polymers

**DOI:** 10.3762/bjnano.8.138

**Published:** 2017-07-04

**Authors:** Hsien-Yeh Chen

**Affiliations:** 1 Department of Chemical Engineering, National Taiwan University, Taipei 10617, Taiwan

**Keywords:** multifunctional, polymer coating, surface modification, surface patterning, vapor deposition

## Abstract

Vapor-deposition processes and the resulting thin polymer films provide consistent coatings that decouple the underlying substrate surface properties and can be applied for surface modification regardless of the substrate material and geometry. Here, various ways to structure these vapor-deposited polymer thin films are described. Well-established and available photolithography and soft lithography techniques are widely performed for the creation of surface patterns and microstructures on coated substrates. However, because of the requirements for applying a photomask or an elastomeric stamp, these techniques are mostly limited to flat substrates. Attempts are also conducted to produce patterned structures on non-flat surfaces with various maskless methods such as light-directed patterning and direct-writing approaches. The limitations for patterning on non-flat surfaces are resolution and cost. With the requirement of chemical control and/or precise accessibility to the linkage with functional molecules, chemically and topographically defined interfaces have recently attracted considerable attention. The multifunctional, gradient, and/or synergistic activities of using such interfaces are also discussed. Finally, an emerging discovery of selective deposition of polymer coatings and the bottom-up patterning approach by using the selective deposition technology is demonstrated.

## Review

### Introduction

Vapor-based processes of polymer coating/deposition combine many unique attributes in a dry, solvent-free process, and the deposition protocols as well as the resulting coatings are mostly applicable to a wide range of substrate materials [1]. In addition, the vapor deposition process typically provides excellent coating fidelity, i.e., the resulting polymer coatings are conformal with respect to micrometer- or nanometer-sized topology of the substrate surface. These unique characteristics are due to the absence of dewetting effects [2], which can make the coatings bridge and buckle. In contrast, dewetting is often encountered in the case of solution-based polymer coatings [3]. Vapor-deposited polymer coatings are widely discussed in interfacial engineering and surface modification technologies for surfaces/devices with sensitive and miniaturized patterns or structures [4–5]. Furthermore, vapor-deposited polymers provide defined chemical control and/or precise accessibility to the linkage with functional molecules at the coating interface. The thrilling developments of such functional activities have recently shown promise to create multiple surface functionalities or gradients that account for the previously mentioned attributes while also rendering the concurrent display of multiple functions and/or synergistic activities to respond to sophisticated microenvironments [6–9].

This review first discusses recent developments in vapor-based polymer deposition and emphasizes the ability to deposit polymers with spatially controlled structures/patterns on the surfaces of substrates regardless of the substrate materials and geometry, i.e., 2D flat substrates or 3D complex substrates. Next, the creation of multiple or gradient structures/patterns on the polymers provides an interfacial template with multifunctional reactivity and gradient information for multifunctional or directional activities. Then, the emerging discovery of the selective deposition of polymer coatings is discussed. This report highlights relevant works and advances by the researchers in the field and is not intended to comprehensively cover the literature from the entire field. Finally, current technological challenges and potential future directions are suggested according to the opinion of the author.

### Structuring of conventional 2D surfaces

**Figure 1:**
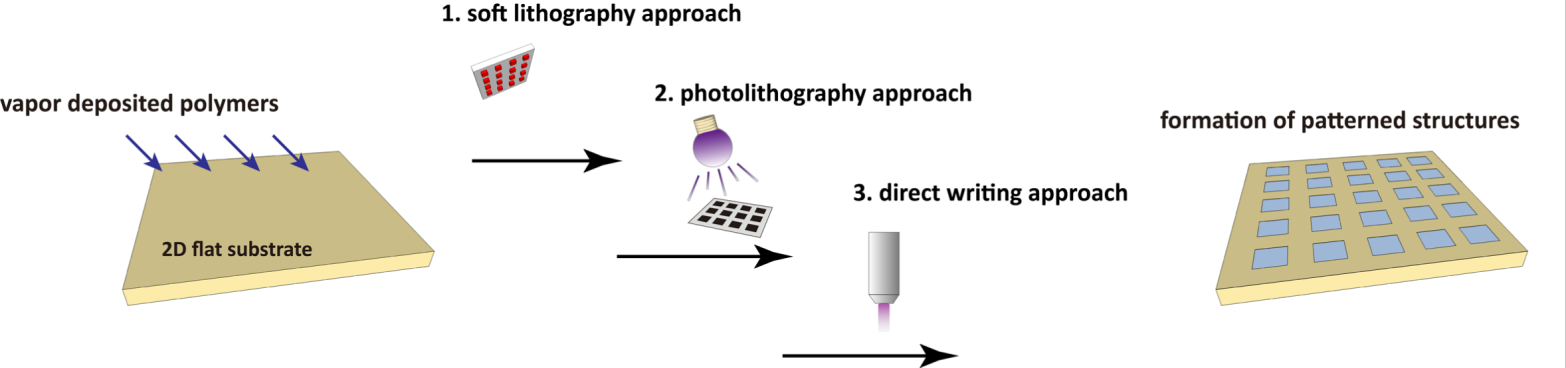
A scheme of creating surface patterns/structures on flat substrates that are modified by vapor-deposited polymer coatings. The patterning methods include soft lithography, photolithography, and direct writing approaches.

Over the past decades, extensive effort has been made and successes have been achieved to create topological surface patterns based on light [10], electrons [11], ion beams [12], X-rays [13], or manipulation of atomic beams [14]. Also, printing methods with elastomeric stamps or replica structures to transfer a material from a solution onto a surface, which are collectively related to imprinting lithography [15–16] or soft lithography [17–18], were developed. Thus, the early developments of the patterning and structuring technologies for vapor-based coatings largely depend on adaptation from these lithographical approaches (Figure 1). A DNA array was fabricated in a photolithographical liftoff process on a vapor-deposited (chemical vapor deposition, CVD) poly-*p*-xylylene surface, and the resulting array surface showed excellent uniformity with reduced array-to-array variation [19]. Vapor-phased plasma polymerization to prepare polyacrylic acid has also used to pattern and functionalize microfluidic devices based on wet and dry etching techniques [20]. Combining plasma polymerization and lithographical processes has also been used for the pattern formation of polyethylene glycol (PEG)-like polymer derivatives to guide fibroblast attachment [21]. A photodefinable polymer of poly(4-benzoyl-*p*-xylylene-*co*-*p*-xylylene) was synthesized by CVD, and a combined soft lithographical and UV light process was performed to create the microstructures of PEG hydrogels [22]. In a separate report, this photodefinable polymer was used to pattern protein molecules using a photomask-assisted lithographical approach [23]. Recently, surface patterns were enabled via light-induced thiol-ene/thiol-yne reactions on a poly(4-vinyl-*p*-xylylene-*co*-*p*-xylylene) surface and a poly(4-ethynyl-*p*-xylylene-*co*-*p*-xylylene) surface, respectively. Various substrates were successfully verified for the coating and patterning modifications: metal (silver, titanium, stainless steel), polystyrene (PS), poly(methyl methacrylate) (PMMA), silicon, glass, poly(dimethylsiloxane) (PDMS), and poly(tetrafluoroethylene) (PTFE) [24]. Microcontact printing (μCP) is a commonly exploited technique that uses a PDMS elastomer to stamp patterns of reactive substances on mostly flat surfaces [17]. It is also widely adopted for the confinement of pattern formation on vapor-deposited coating surfaces. For example, surface patterns were created on a CVD-deposited pentafluorophenol ester-functionalized poly-*p*-xylylene coating by μCP with the use of a PDMS elastomeric stamp, and line patterns of functional biotin molecules were formed with stability up to seven days at room temperature. In the same work, spatial control of the cell attachments and patterns were further produced via the biotin/streptavidin conjugation and subsequently immobilized by the cell-binding antibody [25]. A more delicate pattern formation was generated by combining the μCP technique and the supramolecular nanostamping (SuNS) [26] technology on another vapor-deposited poly(4-formyl-*p*-xylylene-*co*-*p*-xylylene) coating surface, and patterns of DNA molecules were resolved with sizes down to 100 nm. The combination of SuNS with the vapor deposition process enables the extension of the nanopatterning protocols to a range of different substrates, and the nanopatterns were demonstrated on polystyrene, acrylic and PDMS in this work [27]. The aforementioned photolithographical or soft-lithographical methods are simple and straightforward to perform. However, because of the limitation of applying a photomask or an elastomeric stamp, these techniques are mostly limited to flat substrates. The reduced pattern fidelity is resolved from the wider distance of the surface from the photomask or elastomeric stamp on a non-flat or curved surface [23,28–30].

### Structuring approaches not limited to flat surfaces

**Figure 2:**
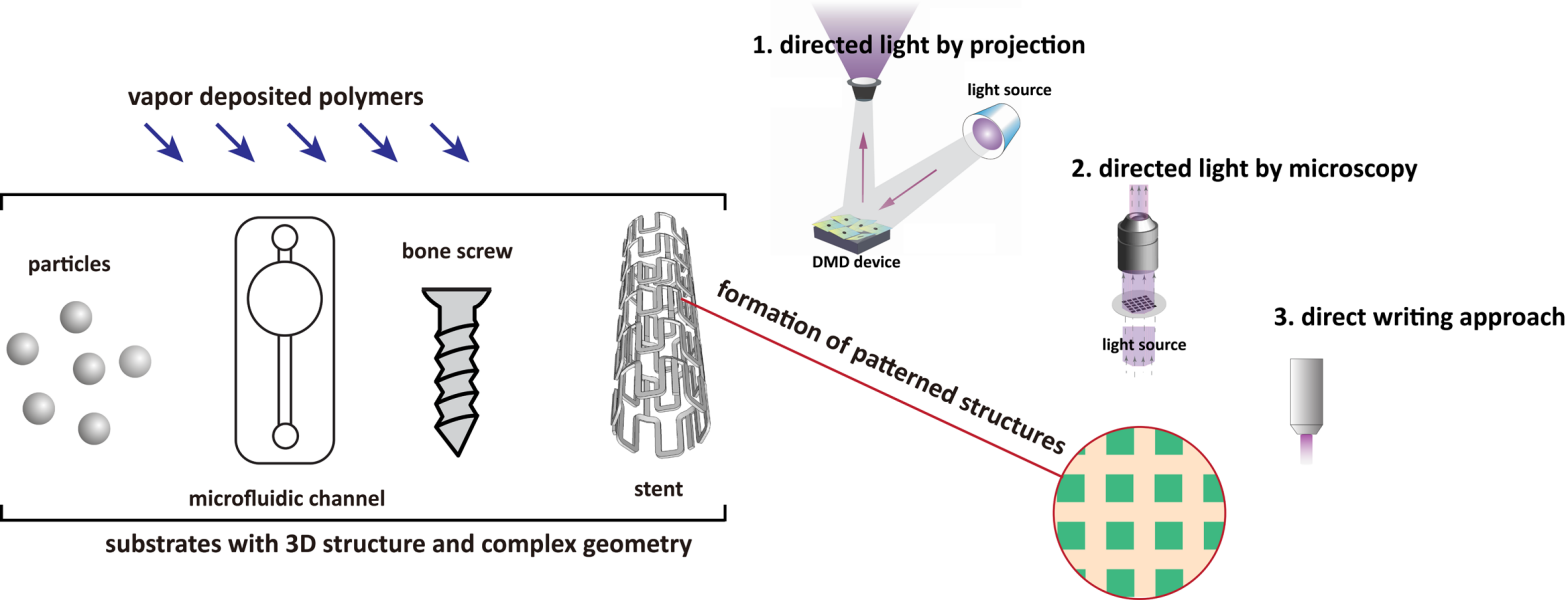
Schematic illustration of creating surface patterns/structures on substrates vapor-coated with polymers with 3D structure and complex geometry. The patterning methods include maskless approaches by light-directed projection, light-directed microscopy and direct writing.

Because vapor polymerization/deposition has the advantage of conformal coverage of substrates, the vapor-phase polymers are freely accessible to deposit on micro- and nano-structured surfaces, curved surfaces, confined microfluidic channels, 3D structures, and substrates with complex geometry [3,31–32]. Although an alternative approach combining vapor deposition of polymers on curved substrates (instead of spin-coating) and a flexible mask to generate polytricosadiynoic acid and poly(4-vinylpyridine) patterns on curvatures has been shown with a conventional lithographic technique [33]. The creation of patterned structures on such non-flat substrates currently requires means different from photomasks or an elastomer stamps to spatially control the modification and construct localized pattern structures, as illustrated in Figure 2. Direct and maskless approaches to apply a patterning at a localized position are attempted by direct electron beam (e-beam) lithography on vapor-deposited PPMA coatings, and 200 nm-sized features were obtained on the vapor-deposited poly(propargyl methacrylate) (PPMA) films [34]. Direct writing using a two-photon laser was also demonstrated on poly(*p*-xylylene) to fabricate 3D nano-/microstructures [35]. Similarly, direct writing using a scanning probe microscopy-based nanolithographic technique (dip-pen nanolithography, DPN) was used to deliver chemical substances with submicrometer features on a wide range of poly(*p*-xylylene) deposited substrates [36]. An array of micro-sized plasma was also used as a maskless method to generate the surface patterning of poly(ethylene oxide) coatings on substrates [37]. An effective maskless approach using directed UV light, for which the light passes through a previously patterned microscopic lens or is projected through a digital micromirror device, was performed to create defined patterns on vapor-deposited poly(*p*-xylylene) surfaces of curved microcolloids [38], microfluidic channels [39], complex stent devices [40–42], and intraocular lens (IOL) devices [43]. Jet deposition was used to prepare a poly(*p*-xylylene) coating under atmospheric conditions and enabled the possibility of direct patterning/writing during the vapor deposition process [44]. A patterning mask made of colloidal crystals has also been demonstrated for the vapor deposition of polymers without requiring photolithographic processes or a stamp [45]. Although most of these techniques remain hampered by the limited resolution of the patterns, they have elegantly contributed to major technological breakthroughs to enable several patterning processes and localized surface modifications on non-flat surfaces for electronics and biotechnology.

### Multifunctional structures

**Figure 3:**
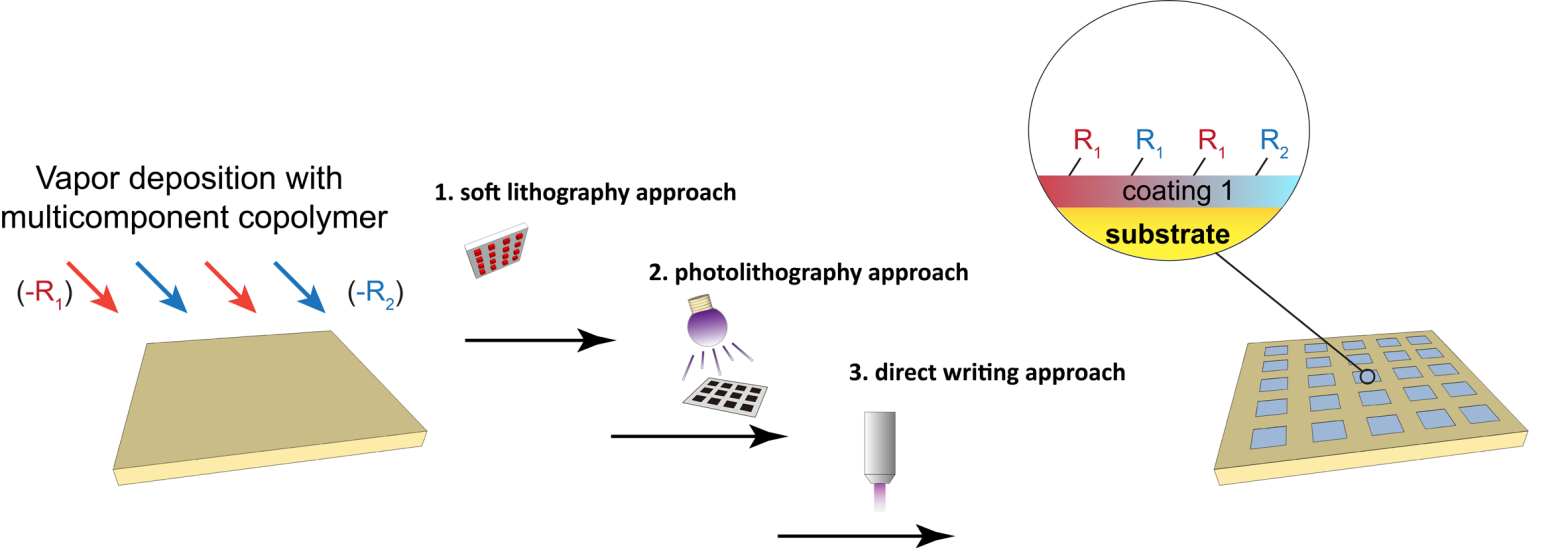
Schematic illustration of creating chemically and topographically defined interfaces with multifunctionality on substrates vapor-coated with polymers. An approach by using multicomponent copolymer coating is demonstrated.

**Figure 4:**
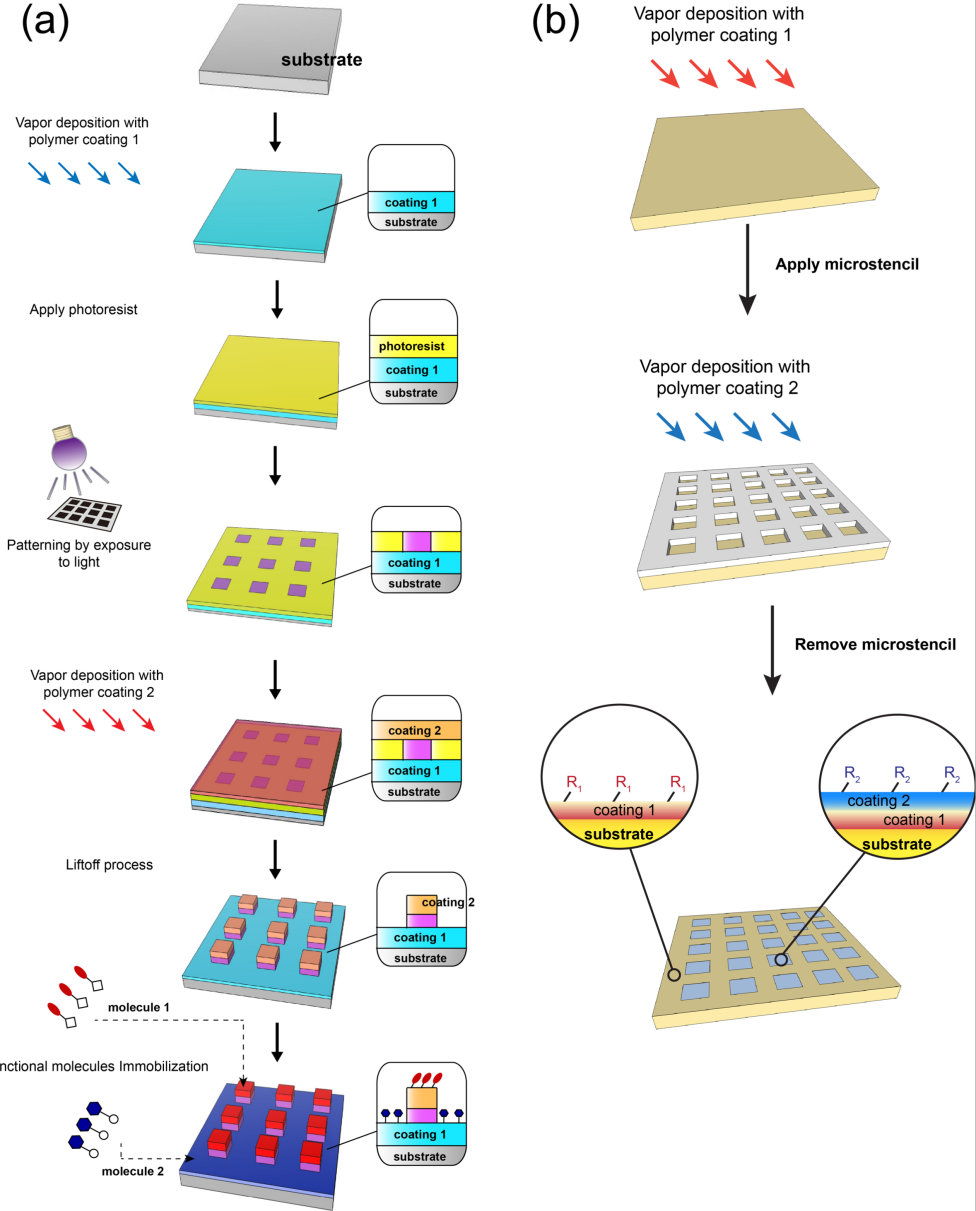
Schematic illustration of creating chemically and topographically defined interfaces with multifunctionality on substrates vapor-coated with polymers. (a) A photolithographic process is performed to prepare chemically and topographically defined surface microstructures on layered functional coatings, and a concept is shown to immobilize multiple functional molecules at corresponding areas. (b) Patterning/structuring on layered polymer coatings by a vapor-assisted micropatterning in the replica structure (VAMPIR) technique, in which a microstencil is exploited during the vapor deposition process. Reproduced with permission from [56], copyright 2014 Wiley-VCH.

The early developments focused on the fabrication of surface patterns and structures with the same physical properties as that the bulk material, interfacial coating materials, patterning processes, and the aspect ratio of formed surface patterns and structures. In addition, the surface chemistry of such patterns and structures, i.e., chemically and topographically defined interfaces, has recently attracted considerable attention, and multifunctional and/or synergistic activities of using such interfaces were successfully demonstrated. The performed approaches were (i) synthesis/deposition of multicomponent copolymers, which contain two or more addressable functional groups, during the surface modification process for substrates, where the multifunctional patterns/structures were formed by subsequently exploiting the aforementioned patterning process (Figure 3), and (ii) an integrated patterning processes of layered depositions of different functional polymer films; the hierarchical structure of the outer layer and exposed underneath layers forms the multifunctional interface (Figure 4). Vapor-based multicomponent copolymers can be synthesized through CVD in one step by introducing independent monomers into the polymerization chamber to form a multi-phasic reactive species (monomer vapors). The copolymerization processes spontaneously occur when the multicomponent copolymer coatings form on substrates [41,46–47]. A wide range of functionalities was demonstrated: combinations of active esters, carbonyls, amino groups, photoactive benzoyls, maleic derivatives, vinyl and alkyne, and aldehydes. Specific and orthogonal reactions were performed to conjugate various molecules, and multifunctional and/or synergistic activities were demonstrated for many applications [9,48–52]. In order to form chemically and topographically defined patterned structures with multifunctional activities and following approach (i), a poly(*p*-xylylene) copolymer that contained both alkyne and pentafluorophenyl ester functionalities was synthesized via CVD copolymerization. This copolymer was used to co-immobilize the cyclic arginine–glycine–aspartic acid (cRGD) adhesion peptide and epidermal growth factor (EGF) in patterned areas via μCP [51]. Another similar copolymer system, which contained methyl propiolate and maleimide moieties, was also synthesized via CVD copolymerization. The concurrently immobilized μCP-patterned PEG and Cys–Arg–Glu–Asp–Val (CREDV) peptide showed the synergic anti-fouling property and preferentially enhanced attachment of endothelial cells in such patterned areas [50]. In another report, a multifunctional coating was realized via CVD copolymerization to deposit a poly(*p*-xylylene) copolymer, which contained distinct *N*-hydroxysuccinimide (NHS) ester and benzoyl functionalities. The copolymer provided accessibility to the NHS ester–amine coupling reaction and the photochemically induced benzophenone crosslinking reaction. These reactions were confined in selected areas using a combination of μCP and a photomask [53]. Meanwhile, approach (ii) was realized with the layered deposition of polymer coatings with one separate functionality for each coating layer. A multifunctional surface containing “PEG-like” and “non-PEG-like” regions has been created by asymmetric glow discharge plasma polymerization [54]. The multifunctional interfaces with pattern structures were demonstrated by separately depositing alkyne-functionalized poly(*p*-xylylene) and aldehyde-functionalized poly(*p*-xylylene) in selected areas using a vapor-assisted micropatterning in the replica structure (VAMPIR) technique [55–56]. The resulting multifunctional patterned surface could spatially direct a combination of Huisgen cycloaddition and carbonyl–hydrazide coupling in a sequentially devised immobilization procedure [52]. A similar sequential immobilization of molecules on defined areas was also performed on a layered coating of propiolate-functionalized poly(*p*-xylylene) and alkyne-functionalized poly(*p*-xylylene), for which VAMPIR was also applied to pattern the layered surface. Two-step click reactions were accessible by using different reactivities of activated and non-activated alkynyl groups towards the azide groups [57]. The idea of using two-step click reactions with approaches (i) and (ii) was also demonstrated by depositing alkyne/maleimide-functionalized poly(*p*-xylylene) copolymer or alkyne-functionalized poly(*p*-xylylene) homopolymer, and multifaceted surface patterns were obtained via route-controlled click reactions with μCP or a photomask [49].

### Gradient structures

Surface gradients represent an advanced surface modification tool to exert gradient activities and/or communicate with the microenvironment using gradually altered cues. Such gradients include physical properties such as the wettability, thickness, dielectric constant, temperature, and morphology and various chemical compositions [58–65]. Because of the challenges in fabrication processes, gradients are often generated with solution-based technology. Limitations remain for the ongoing technologies, for example, the lack long-term stability due to degradation or desorption from the modified surface [66–67], or hardly predictable biological outcomes of interactions between the biological environment and the materials interfaces [60]. Moreover, widely used laminated/layered constructs are limited through the boundary discontinuities across layers of dissimilar materials or properties [68]. In addition to the current solution-based techniques, vapor-deposited polymer coatings have been developed to create surface-gradient patterns and provide advantages with precisely controlled chemical or biological compliance without restrictions in selecting substrate materials and geometries [69]. By using corona discharge treatment with gradually increasing power, the density of PEG was controlled with gradients to guide protein adsorption and platelet adhesion [70]. By also controlling polyatomic ion deposition to linearly increase the C_3_F_5_^+^ ion fluence across polymer, metal, and silicon substrates, a hydrophobicity gradient was formed along the treatment direction [71]. The chemical gradients of hydrophobic octadiene to a more hydrophilic acrylic acid were produced via plasma polymerization, and the surface was found effective for cell pluripotency against mouse embryonic stem cells [72]. A plasma-polymerized surface with gradient amino functionality was demonstrated to generate density gradients of individual gold (Au) and silver (Ag) nanoparticles on the surfaces [73]. Poly(*p*-xylylene) surfaces with continuously and counter-currently distributed functionality gradients of active carbonyls and amines were synthesized by diffusing individual monomer vapor from the opposite direction during the CVD copolymerization process [8]. In an extended work, another version of the gradient copolymer containing aldehydes and amine gradients was generated, and a subsequent cell-culture study showed that cell-signaling adenovirus was correlated along the copolymer gradients [74]. A similar combinatorial approach has also been demonstrated to generate poly(diethylaminoethylacrylate) and poly(dimethylaminomethylstyrene) gradients using an initiated CVD system [75]. The route-controlled click reactions, including a thiol-yne reaction and a copper-free alkyne/azide click reaction, were enabled to create continuous and reverse gradients on a CVD deposited poly[(4-methylpropiolate-*p*-xylylene)-*co*-(*p*-xylylene)] surface. The two-click reactions were employed to co-immobilize fibroblast growth factor 2 (FGF-2) and bone morphogenetic protein 2 (BMP-2) and established reverse gradient distributions of the FGF-2 and BMP-2. Furthermore, these two growth factors gradients have demonstrated the corresponding biological activities toward both proliferation (FGF-2) and osteogenic differentiation (BMP-2) for adipose-derived stem cells [76].

### Selective deposition

The aforementioned methods rely on physical means to obtain spatially controlled surface modifications and patterned structures. A simpler approach is the selective inhibition of the vapor deposition/polymerization process on substrates, i.e., the polymer coatings are either deposited or not on substrates because of the chemistry below the substrate surface. The mechanism of the polymer deposition selectivity is not conclusive. The inhibition of polymer deposition is believed to occur because of the high surface energy of the substrate, which neutralizes the reactive monomer species that are adsorbed on the substrate surface and prevents further initiation and propagation of the polymerization reaction. For example, non-substituted *p*-xylylene and chlorine-substituted *p*-xylylene (monomers of two types of poly-*p*-xylylenes, which are commercially named parylene™ N and parylene™ C, respectively) were found to deactivate on several high-energy surfaces of several transition metals such as iron, copper, silver, platinum, and the salts of these metals. The monomer deactivation inhibits the deposition of parylene™ N and parylene™ C on these high-energy metal surfaces. The degree of selectivity (there exists an upper limit, where deposition will commence and the relative selectivity is lost) is different for different metal surfaces and correlates with the deposition rate [77]. Based on the discovery, applications have been demonstrated to generate Nomarski poly(*p*-phenylene vinylene) (PPV) patterns from selectively deposited parylene™ N on surfaces with photolithographically fabricated iron structures (inhibitors) [78]. A required pore-sealing process for porous dielectrics was also performed using selectively deposited parylene™ N to avoid the deposition on sub-45 nm copper nodes [79]. The copolymer poly(4-vinyl pyridine-*co*-divinyl benzene) was selectively deposited on a chromatography paper with screen-printed copper(II) chloride patterns [80]. A comprehensive study further examined the deposition of a wide range of functionalized poly(*p*-xylylenes) on high-energy metal surfaces. The study found that the deposition selectivity might have been compromised, and a possible explanation may be that neutralization occurred between the oxygen or nitrogen from the side groups of the functionalized *p*-xylylenes and the high-energy metal substrates by attraction interactions. In contrast, an inhibitor surface experiences neutralization and deactivation at the free radicals for halogen- or non-substituted *p*-xylylenes. A continuum of deposition and polymer chain propagation can thus proceed for the case of functionalized *p*-xylylenes [81]. The compromised selectivity was recently reactivated by supplying electrical energy to the (conducting) substrates. The deposition selectivity was enhanced by increasing the transition of the surface energy instead of relying on native surface energy of the substrates. In other words, effective selectivity for the deposition of nonfunctional poly(*p*-xylylene) has been achieved, and the family of functionalized poly(*p*-xylylene) is now manageable [82].

## Conclusion

As more stringent specifications are required for designing the surface properties of prospective materials, and in addition, the development of new devices is pursued with complicated geometries and minimized sizes, the surface properties of such materials/devices now also require a more defined and flexible presentation of the chemical functionalities (e.g., multifunctional or gradient distribution) and the precise confinement of these chemical conducts in relevant locations of interest. The vapor deposition process and the resulting thin polymer films provide consistent coatings, which decouple the underlying substrate surface properties and can be applied for surface modification on most of the substrate geometry and materials (with the exception for the case of selective deposition on transition metals and charged surfaces). Because of the well-established and available photolithography and soft lithography techniques, promising patterned surface structures have been created. Attempts were conducted to produce patterned structures on non-flat surfaces. However, techniques such as directed light or direct writing approaches currently have limitations regarding the resolution and cost. Thus, new techniques are developed to push the resolution limit and decrease the cost for the possibility of practical applications. An emerging question may have arisen because vapor-deposited species are free of the geometrical limits of the substrate, i.e., vapor species can deposit on curvatures and confined microgeometries. However, the patterning techniques are only available to perform on accessible surfaces but not in overhanging or sealed surfaces. For example, the problem of how to pattern and structure an internal lumen of a microchannel while the surface can be modified using vapor-deposited polymers remains unsolved and is encouraged for dedicated work from researchers in this field. A more general problem of the vapor deposition process us that the process mostly requires vacuum conditions to protect the reactive vapor species (monomers) from side reactions, which hampers the application as a continuous mass production process. A vacuum-free method [44] may solve the problem, but several engineering works and system parameters for other vapor deposition systems must be optimized. Nevertheless, vapor-deposited polymers offer unrivaled coating fidelity and precise control over the surface chemistry. The integration of polymer coatings and patterning technologies results in interface properties that account for both chemically and topologically defined properties, which is a promising tool to design prospective multidisciplinary materials. More applications using these technologies are only limited by imagination.
